# Intracellular prostaglandin E2 contributes to hypoxia-induced proximal tubular cell death

**DOI:** 10.1038/s41598-021-86219-w

**Published:** 2021-03-29

**Authors:** Coral García-Pastor, Selma Benito-Martínez, Ricardo J. Bosch, Ana B. Fernández-Martínez, Francisco J. Lucio-Cazaña

**Affiliations:** 1 Departamento de Biología de Sistemas, Universidad de Alcalá, Alcalá de Henares, Madrid, Spain; 2 Departamento de Biología, Universidad Autónoma de Madrid, 28049 Madrid, Spain

**Keywords:** Cell biology, Drug discovery, Physiology, Diseases

## Abstract

Proximal tubular cells (PTC) are particularly vulnerable to hypoxia-induced apoptosis, a relevant factor for kidney disease. We hypothesized here that PTC death under hypoxia is mediated by cyclo-oxygenase (COX-2)-dependent production of prostaglandin E_2_ (PGE_2_), which was confirmed in human proximal tubular HK-2 cells because hypoxia (1% O_2_)-induced apoptosis (i) was prevented by a COX-2 inhibitor and by antagonists of prostaglandin (EP) receptors and (ii) was associated to an increase in intracellular PGE_2_ (iPGE_2_) due to hypoxia-inducible factor-1α-dependent transcriptional up-regulation of COX-2. Apoptosis was also prevented by inhibitors of the prostaglandin uptake transporter PGT, which indicated that iPGE_2_ contributes to hypoxia-induced apoptosis (on the contrary, hypoxia/reoxygenation-induced PTC death was exclusively due to extracellular PGE_2_). Thus, iPGE_2_ is a new actor in the pathogenesis of hypoxia-induced tubular injury and PGT might be a new therapeutic target for the prevention of hypoxia-dependent lesions in renal diseases.

## Introduction

It is well established that tubular hypoxia is a relevant factor for both acute and chronic kidney disease^1,2^ Proximal tubular cells (PTC) are highly active in terms of oxygen consumption because of their energy-demanding activities of reabsorption and, consequently, they are vulnerable to hypoxia^3^. It has been shown that apoptosis plays a relevant role in cultured PTC exposed to hypoxia^4–6^, but the signalling pathways responsible for the activation of the apoptotic machinery have not been investigated. We and others have found that prostaglandin E_2_ (PGE_2_), an important lipid mediator of numerous physiological and pathological processes in the kidney, plays a relevant role in the signalling leading to PTC apoptosis upon treatment with cisplatin^7^, albumin^8^ or leptin and gentamycin^9^. In all the instances, the increase in PGE_2_ was found to be dependent on enhanced expression of cyclo-oxygenase-2 (COX-2), an inducible enzyme which, together with COX-1, is the rate-limiting step in the synthesis of prostaglandins. In the human kidney, basal COX-2 expression is less intense than that of COX-1. COX-2 has been identified in podocytes and parts of the loop of Henle and renal vasculature under physiological conditions^10^. In pathological states, however, COX-2 immunoreactivity may be found in many more cell types within the kidney including PTC and possibly contributes to renal injury^11^. Interestingly, hypoxia increases the expression of COX-2 and/or the production of PGE_2_^12–17^ in many cell types. In fact we have previously found that hypoxia enhances the intracellular content in PGE_2_ as well as its release to the extracellular medium^18^. Therefore, it is theoretically possible that PGE_2_ mediates the apoptotic effect of hypoxia on PTC.

Most studies on PGE_2_-dependent apoptosis have essentially been focused on mechanisms involving extracellular PGE_2_ because it is widely accepted that PGE_2_ exerts its biological effects through activation of plasma membrane spanning G-protein coupled PGE_2_ receptors (E series prostaglandin receptors EP1-4)^19^. Nevertheless, in some models, *intracellular* PGE_2_ (iPGE_2_) is relevant in apoptotic cell death^7,20–22^. This implies that, to induce apoptosis, PGE_2_ has to reach the intracellular medium again and activate a subset of EP receptors, which are located inside the cell (iEP receptors). Because the task of capturing PGE_2_ is mainly done by the prostaglandin uptake transporter (PGT)^23–26^, PGT inhibition results in prevention of iPGE_2_-mediated apoptotic cell death^7^.

Taking into account this background, in the present work, we studied whether a COX-2-dependent increase in iPGE_2_ levels mediates hypoxia-induced apoptosis in PTC.

## Methods

### Reagents

AG1478, Bromocresol green (BG), PGE_2_, AH6809, GW627368X, crystal violet, trypan blue solutions, Bromosulfophthalein (BrS), 3-(5′-Hydroxymethyl2′-furyl)-1-benzyl indazole (YC1), and actinomycin D were purchased from Sigma (St. Louis, MO, USA). Z-VAD-FMK and celecoxib were from Calbiochem (Darmstadt, Germany) and Cayman Chemical (Ann Arbor, MI, USA), respectively. TriReagent was purchased from Vitro (Madrid, Spain), and PVDF membranes and Western blotting luminol reagent were acquired from Santa Cruz Biotechnology (Santa Cruz, CA. USA). ProLong Gold antifade reagent with 4,6-diamidino-2-phenylindole (DAPI), annexin-V–FITC (fluorescein isothiocyanate)/propidium iodide (PI) apoptosis detection kit and 2′,7′-dichlorofluorescein diacetate (DCFH-DA) probe were purchased from Invitrogen (Carlsbad, CA, USA), and Molecular Probes (Oregon, USA), respectively. Antibodies were obtained from the following sources: anti-PGE_2_ and anti-COX-2 antibodies were from Abcam (Cambridge, UK); anti-Bax and anti-Bcl-2 were from Santa Cruz Technologies (Santa Cruz, CA. USA); anti-HIF-1α antibody and α-rabbit-Alexa-Fluor 488 were from BD Biosciences (Palo Alto, CA, USA) and Invitrogen (Carlsbad, CA, USA), respectively; anti-β-actin and rabbit anti-mouse IgG peroxidase conjugate were purchased from Sigma (St. Louis, MO, USA).

### Cell culture and experimental conditions

Human proximal tubular HK-2 cells were purchased from the American Type Culture Collection (ATCC) (Rockville, MD, USA). Cells were maintained in 5% CO_2_ at 37 ºC in DMEM/F12 supplemented with 10% fetal bovine serum (FBS), 1% penicillin/streptomycin/amphotericin B and 1% glutamine (Invitrogen. Carlsbad, CA, USA) and 1% insulin-transferrine-selenium (ThermoFisher. Grand Island, NY, USA). In all experiments, cells were plated at 70–90% confluence, and when completely attached, they were cultured under hypoxic (1% oxygen) or normoxic conditions (21% oxygen). Hypoxia experiments were performed in an In vivo200 hypoxia workstation (Ruskinn Technology, West Yorkshire, United Kingdom). For hypoxia/reoxygenation experiments, cells were subjected to hypoxia for 24 h, and, thereafter, cells were incubated in normoxic conditions for up to 3 h (reoxygenation period).

### Immunofluorescence analysis of iPGE_2_ and Western blot analysis of COX-2 and HIF-1α

Cells for immunofluorescence analysis and Western blot analysis were respectively split on glass coverslips (4 × 10^4^ cells/glass coverslip) or into six-well plates (15 × 10^4^ cells/well) and incubated as described in “Results”. Then, immunofluorescence, and immunoblotting analysis were performed essentially as described previously^7^. Antibody working dilutions were: 1/50 for PGE_2_, 1/1000 for COX-2/HIF-1α/α-rabbit-Alexa-Fluor 568 and 1/5000 for β-actin. Immunofluorescence detection was performed using a Leica SP5 confocal microscope (Leica Microsystems, Wetzlar, Germany), through the Confocal Microscopy Service (ICTS ‘NANBIOSIS’ U17) of the Biomedical Research Networking Centre on Bioengineering, Biomaterials, and Nanomedicine (CIBER-BBN at the University of Alcalá, Madrid, Spain) (http://www.uah.es/enlaces/investigacion.shtm).

### Transient transfection

Transient transfection with siRNA PGT (Santa Cruz Biotechnology, Santa Cruz, CA), the mammalian expression vector pcDNA3 containing the cDNA of the wild-type human 15-hydroxy-prostaglandin dehydrogenase (p15-PGDH) or the luciferase reporter plasmid constructs for human COX-2 phPES2-Luc, human hypoxia response element (HRE) p9HIF1-Luc and *R. reniformis* pRL-CMV (Promega, Madison, WI) and determination of luciferase activity were performed as described elsewhere^27,28^.

### Cell count and cell/nucleus morphology

The number of adherent cells was determined spectrophotometrically with a modified crystal violet staining method^29^. To detect evidence of apoptosis, cell morphology was observed using phase-contrast microscopy. Cell nuclei were visualized after DNA staining with DAPI as previously described^30^. Typical apoptotic morphology that was examined included cellular shrinkage, nuclear condensation and fragmentation, and formation of apoptotic bodies. For quantification, six fields were examined in each experimental condition in a blind manner to estimate the percentage of nuclei with apoptosis-like appearance.

### Flow cytometry apoptosis assay and cell viability assay by trypan blue dye exclusion test

Adherent cells to the plate were detached by trypsinization and, together with the detached cells previously recovered from the culture medium, were used for assay.

Annexin-V–FITC/PI apoptosis detection kit allowed for flow cytometry detection of apoptotic and necrotic HK-2 cells, as previously described^7^. Early and late apoptotic cells were positive, respectively, to annexin V staining and both PI and annexin V staining. Necrotic cells were only positive to PI and live HK-2 cells showed no staining.

Trypan blue dye exclusion test was used to assess cell viability. Viable cells (white) and dead cells (blue) were counted using a light microscope and a hemocytometer.

### Statistical analysis

Each experiment was repeated at least three times. The results are expressed as the mean ± SEM. They were subjected to a one-way analysis of variance (ANOVA) following by Bonferroni's test for multiple comparisons. The level of significance was set at *P* ≤ 0.05.

## Results

### Hypoxia induces proximal tubular HK-2 cell death

**Figure 1:**
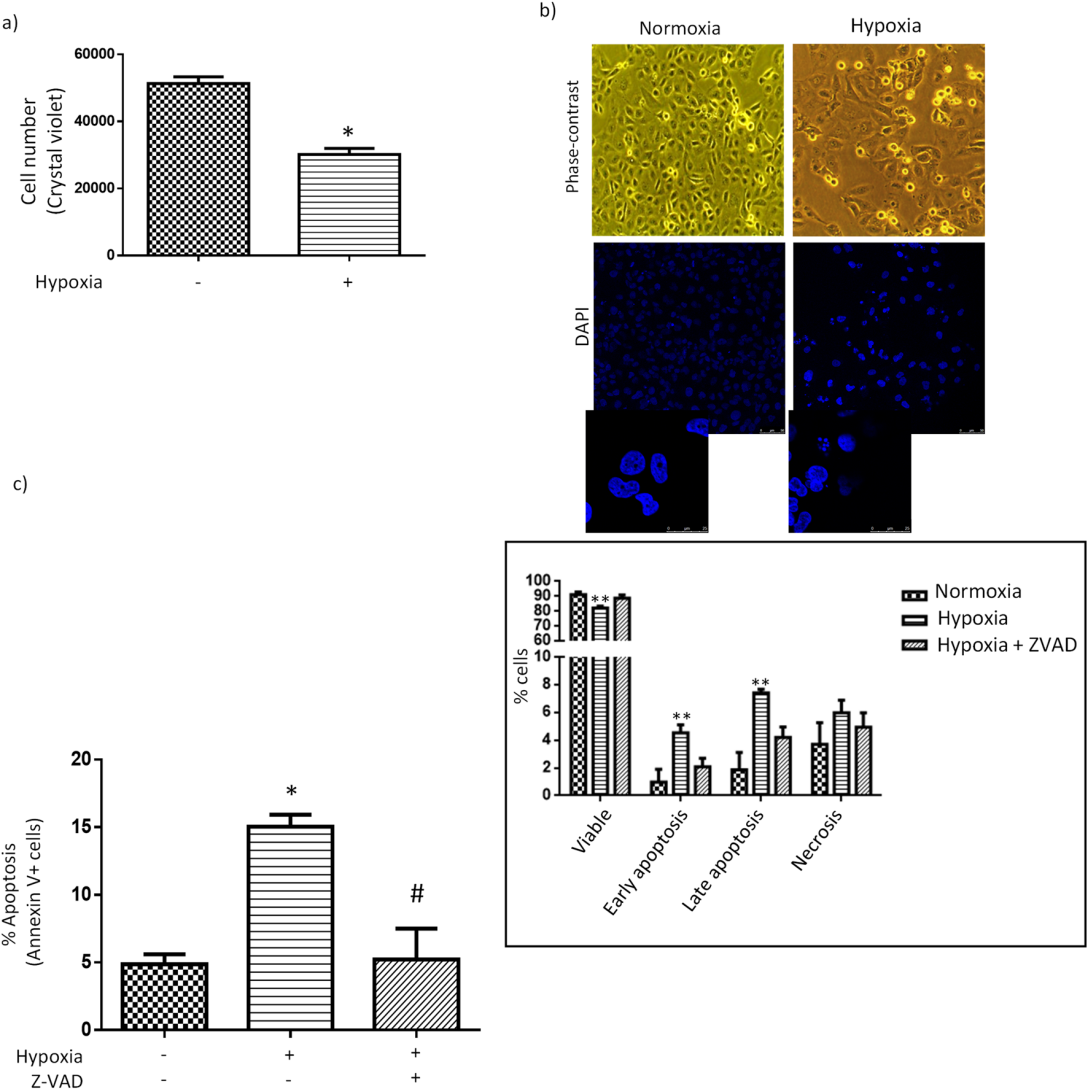
Hypoxia induces proximal tubular HK-2 cell death. (**a**) Diminished cell count (crystal violet assay). (**b**) Morphological characteristics of apoptosis. Representative phase-contrast photomicrographs (upper panel, original magnification, 10×) DAPI staining photomicrographs (lower panel, original magnification, 40×). (**c**) Increase in apoptosis (flow cytometry studies). Annexin V + cells comprise Annexin V+/propidium iodide − cells (i.e. early apoptotic cells with preserved plasma membrane integrity) and annexin V +/propidium iodide + cells (i.e. late apoptotic cells). Inset: Types of cell populations (Annexin V−/  propidium iodide + cells are necrotic cells and annexin V−/ propidium iodide− are cells alive). Results are expressed as percentage with respect to total number of events. General information: (1) Cells were incubated for 24 h in normoxic (21% O_2_) or hypoxic (1% O_2_) conditions, as indicated in “Methods”. Pan-caspase inhibitor Z-VAD-FMK (25 µM) was added 1 h before starting exposure to hypoxia. (2) Microphotographs are representative examples of three independent experiments. Bars and error bars in graphs: Each bar represents the mean ± SEM of 3 different experiments *P < 0.01 vs. control; ^#^P < 0.01 vs hypoxia; **P < 0.01 vs. normoxia and hypoxia + ZVAD.

Hypoxia reduced the number of HK-2 cells, as assessed by crystal violet assay (Fig. 1a), and also induced cell rounding and detachment from the plate (Fig. 1b, upper panel). As expected, hypoxia triggered a model of cell death with clear morphological characteristics of apoptosis (see nuclear staining with DAPI in Fig. 1b, lower panel, left) such as cell shrinkage with significant nuclear condensation, fragmentation, and formation of apoptotic-like bodies. Hypoxia-induced apoptosis was further confirmed by the increase of annexin V-FITC staining, as assessed by flow cytometry, and its prevention with pan-caspase inhibitor Z-VAD-FMK (Fig. 1c). Hypoxia also determined a decrease in the number of viable cells but did not induce statistically significant changes in the number of necrotic cells (Fig. 1c, inset).

### COX-2/iPGE_2_/EP receptor pathway mediates proximal tubular HK-2 cell death induced by hypoxia

**Figure 2:**
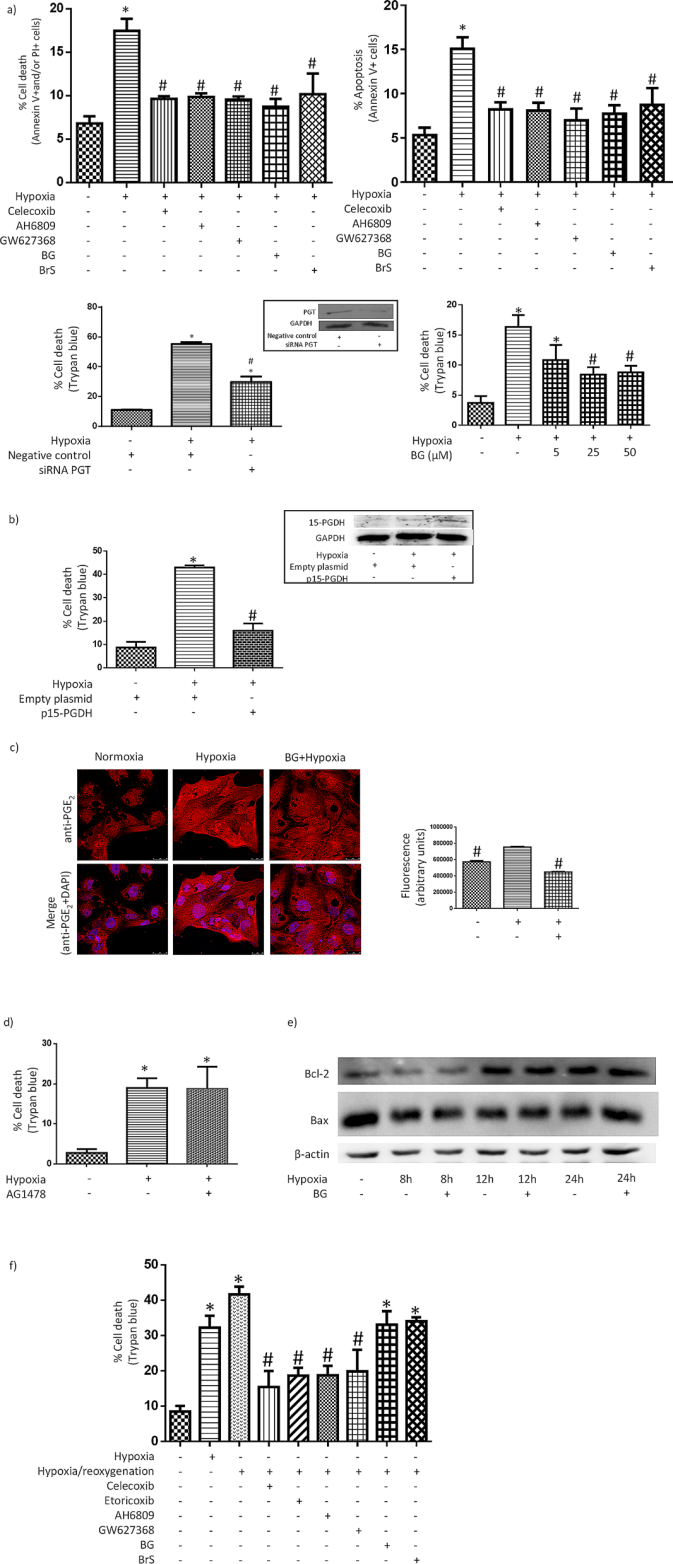
COX-2/iPGE_2_/EP receptor pathway mediates hypoxia-induced proximal tubular HK-2 cell death. (**a**) Prevention of cell death by inhibitors of the pathway. Upper panel: The bars show the percent of total cell death (left) or the percent of apoptotic annexin V + cells (right). Before being exposed to hypoxia, cells were pre-incubated for 1 h with 3 µM celecoxib (COX-2 inhibitor); 10 µM AH6809, (EP1-3 receptors antagonist), 10 µM GW627368X (EP4 receptor antagonist); or with 50 µM bromocresol green (BG) or 25 µM bromosulfophthalein (BrS), two PGT inhibitors. Lower panel: Left: Prevention of cell death by knocking-down PGT though transfection with siRNA (trypan blue dye exclusion test). Inset: Effectivity of the knock down of PGT (Western blot analysis) Right: Concentration dependence of the preventive effect of bromocresol green (BG). (**b**) Prevention of cell death by transfection with a mammalian expression vector containing prostaglandin inactivating enzyme 15-hydroxy-prostaglandin dehydrogenase (15-PGDH) cDNA (trypan blue dye exclusion test). Inset: Expression of 15-PGDH (Western blot analysis) in transfected cells. (**c**) Hypoxia induces a PGT-sensitive increase in iPGE_2_. Left*:* PGE_2_-dependent immunofluorescence alone or merged with nuclear staining with DAPI is shown (original magnification, 40×) Right*:* Quantitative approach to the images presented in left panel using Image J software. (**d**) Inhibitor of EGFR activation AG1478 does not prevent HK-2 cell death EGFR. Cells were pre-incubated for 1 h with 1 μM AG1478 before being subjected to hypoxia. (**e**) PGT inhibitor BG does not modify the expression of Bax and Bcl-2 in HK-2 cells under hypoxia (Western blot analysis). (**f**) Prevention of hypoxia/reoxygenation-induced cell death by inhibitors of the pathway (trypan blue dye exclusion test). Cells were pre-incubated with inhibitors of the pathway and subjected to hypoxia as in (**a**). Then, cells were exposed to normoxia for 3 h. General information: (1) Cells were incubated for 24 h in normoxic (21% O_2_) or hypoxic (1% O_2_) conditions, as indicated in “Methods”. (2) Microphotographs are representative examples of three independent experiments. (3) Solvents of the inhibitors (1μL/mL medium) did not modify the effect of hypoxia on cell death (results are not shown). (4) Western blot analysis: Equal protein was confirmed by probing with an anti-β-actin or an anti-GAPDH antibody. (5) Bars and error bars in graphs: Each bar represents the mean ± SEM of 3 different experiments. *P < 0.01 vs. control; ^#^*P* < 0.01 vs. hypoxia or hypoxia/reoxygenation.

In order to assess the role of the COX-2/iPGE2/EP receptor pathway in hypoxia-induced HK-2 cell death, we first pre-incubated cells with celecoxib, a COX-2 inhibitor^31^; AH6809, an antagonist of EP1, EP2 and EP3 receptors^32^ or GW627368X, an antagonist of EP4 receptor^33^; or with bromocresol green or bromosulfophthalein, both of them inhibitors of PGT^34,35^. PGT was also knocked-down through transfection with siRNA. As shown in Fig. 2a, hypoxia-induced proximal tubular cell death was prevented in all instances. Inhibition of apoptosis by celecoxib indicated that COX-2 plays a relevant role in hypoxia-induced apoptotic cell death (Fig. 2a, upper panel, right). More specifically, the fact that apoptosis was prevented by antagonism of EP receptors bespeaks that apoptosis is mediated by COX-2-dependent production of PGE_2_. On the other hand, it is most likely that iPGE_2_ contributes to hypoxia-induced HK-2 cell death because it was prevented by: (i) inhibition of PGT (Fig. 2a) or (ii) overexpression of 15-PGDH (Fig. 2b), which inactivates iPGE_2_ through its oxidation^25^ (of note, the transfection procedure itself increased the sensitivity of HK-2 cells to hypoxia: cell death was 45–55% for transfection in control conditions compared to 15–20% in non-transfected cells). Further support to the contribution of iPGE_2_ to hypoxia-induced HK-2 cell death are our previous findings that hypoxia determines an increase in the cell content of iPGE_2_^18^ and that this increase was prevented by PGT inhibitors (Fig. 2c).

MAPK signaling pathways may induce apoptosis. Because iEP receptors transactivate epidermal growth factor receptor (EGFR)^36^, which leads to activation of MAP kinases ERK1/2 and p38 in HK-2 cells^30^, we asked whether pre-incubation of HK-2 cells with the inhibitor of EGFR activation AG1478 prevented hypoxia-induced cell death. We found negative results (Fig. 2d) and therefore, it is unlikely that transactivation of EGFR mediates HK-2 cell death under hypoxia.

In several experimental models, the reduction of mitochondrial-derived ATP during hypoxia causes an increase in the pro-apoptotic protein Bax to anti-apoptotic protein Bcl-2 expression ratio, which leads to cytochrome C release into the cytosol, activation of caspase 9, and subsequent cleavage and activation of downstream, effector caspases^37,38^. However, pre-incubation with PGT inhibitor BG in HK-2 cells under hypoxia did not result in changes compatible with an anti-apoptotic shift in the balance between the expression of Bcl-2 and Bax (Fig. 2e), which does not support a role of these proteins in HK-2 cell death under hypoxia.

Following hypoxic exposure, episodes of reoxygenation (ischemia/reperfusion injury) can induce additional cell damage^37^. The “paradox” of reoxygenation injury can be understood taking into account that cells undergo specific changes in enzyme activities, mitochondrial function, cytoskeletal structure, membrane transport, and antioxidant defenses in response to hypoxia, which then collectively predispose to reoxygenation injury^39^. Reoxygenation contributes to acute kidney injury during ischemic stroke, kidney transplantation, circulatory failure or renal and cardiovascular surgery^40^. In this context, the potential therapeutic value of the inhibition of hypoxia/reoxygenation-induced proximal tubular cell death through intervention in COX-2/iPGE_2_/iEP receptor pathway is evident. Therefore, we asked whether iPGE_2_ specifically mediates hypoxia-induced cell death or also mediates hypoxia/reoxygenation-induced cell death (an in vitro model which mimics in vivo renal ischemia/reperfusion injury^41^). As shown in Fig. 2f, cell death (as assessed by trypan blue dye exclusion test) was prevented by COX-2 inhibitor celecoxib as well as by EP receptor antagonists AH6809 or GW627368X. Same than for Fig. 2a, these results suggest that COX-2 plays a relevant role in hypoxia/reoxygenation-induced cell death and, more specifically, that cell death is mediated by COX-2-dependent production of PGE_2_, since it was prevented by antagonism of EP receptors. However, because cell death was not prevented by PGT inhibitors bromocresol green or bromosulfophthalein (Fig. 2f and Supplementary Figure 2d), it is more likely that hypoxia/reoxygenation-induced HK-2 cell death is mediated exclusively by extracellular PGE_2_.

### HIF-1α-dependent transcriptional regulation of COX-2 gene expression contributes to hypoxia-induced increase in iPGE_2_ in proximal tubular HK-2 cells

**Figure 3:**
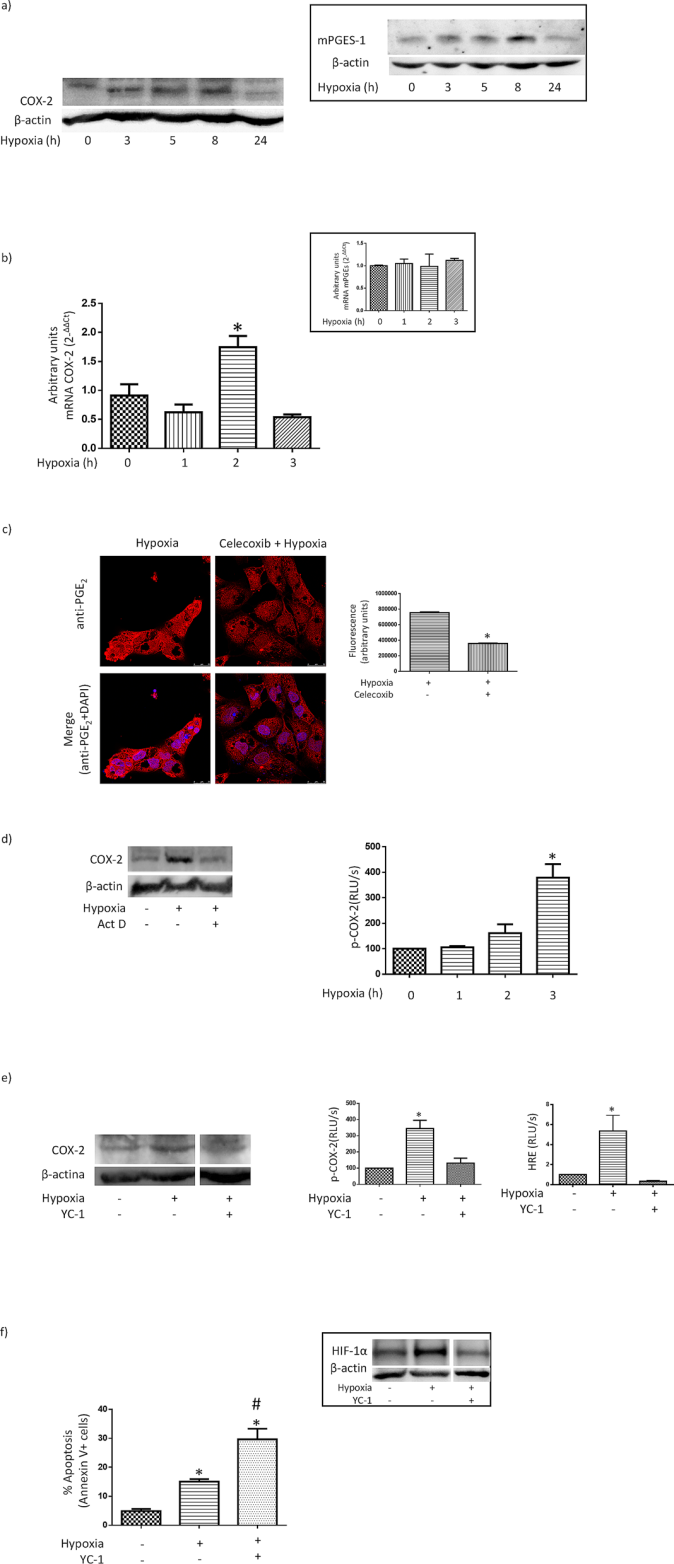
HIF-1α-dependent transcriptional regulation of COX-2 gene expression contributes to hypoxia-induced increase in iPGE_2_ in proximal tubular HK-2 cells. (**a**) Expression of COX-2 protein is transiently increased by hypoxia (Western blot analysis). Inset: Expression mPGES-1 protein is also transiently up-regulated by hypoxia. (**b**) Expression COX-2 mRNA is transiently increased by hypoxia. Inset: Expression of mPGES-1 mRNA is unaffected by hypoxia. (**c**) Prevention by celecoxib, a COX-2 inhibitor, of the increase in iPGE_2_ induced by hypoxia. Cells were preincubated for 1 h with 3 µM celecoxib. *Left:* PGE_2_-dependent immunofluorescence alone or merged with nuclear staining with DAPI is shown (original magnification, 40×). *Right:* Quantitative approach to the images presented in left panel using Image J software. (**d**) Contribution of transcriptional mechanisms. Left: Transcriptional inhibitor actinomycin D (Act. D) prevents hypoxia-induced increase in COX-2 protein expression (Western blot analysis). Cells were pre-treated for 1 h with 1 μg/ml Act. D before being exposed to hypoxia for 3 h. Right: Increase in the activity of a COX-2 reporter construct. (**e**) Inhibitor of HIF-1α YC-1 prevents hypoxia-induced transcriptional COX-2 up-regulation. Cells were preincubated for 1 h with 0.5 µM YC-1. Left: Prevention of COX-2 up-regulation. Cells were exposed to hypoxia for 8 h. Center: Prevention of the increase in the activity of a COX-2 reporter construct. Right: Inhibition of the activity of a HRE-driven reporter construct. Cells were exposed to hypoxia for 8 h. (**f**) HIF-1α inhibitor YC-1 increases apoptotic cell death (flow cytometry studies). Before being exposed to hypoxia, cells were pre-incubated for 1 h with 0.5 µM YC-1. Inset: YC-1 inhibits hypoxia-induced increase in HIF-1α expression (Western blot analysis). General information: (1) Cells were incubated for 24 h in normoxic (21% O_2_) or hypoxic (1% O_2_) conditions, as indicated in “Methods”. (2) Microphotographs are representative examples of three independent experiments. (3) Western blot analysis: Equal protein was confirmed by probing with an anti-β-actin antibody (4) Bars and error bars in graphs: Each bar represents the mean ± SEM of 3 different experiments *P < 0.01 vs. other groups.

PGE_2_ biosynthesis involves the release from membrane glycerophospholipids of arachidonic acid by phospholipase A_2_, followed by the conversion by COX isoenzymes of arachidonic acid to prostaglandin H_2_ and, finally, by the isomerization of prostaglandin H_2_ to PGE_2_ by prostaglandin E synthases such as microsomal PGE synthase-1 (mPGES-1)^19^. We have found that hypoxia-induced apoptosis in HK-2 cells is most likely mediated by a COX-2-dependent increase in the production of PGE_2_ (Fig. 2). Given that COX-2 is an enzyme whose expression may be induced by hypoxia, we hypothesized that hypoxia-induced increase in PGE_2_ production in HK-2 cells might be the consequence of an increase in the expression of COX-2. In order to confirm our hypothesis, we studied (i) the effect of hypoxia on the expression of COX-2 protein and mRNA and (ii) the effect of COX-2 inhibitor celecoxib on hypoxia-induced increase in iPGE_2_. Our results indicated that hypoxia determined in a transient manner transcriptional up-regulation of COX-2 expression (Fig. 3a,b) and that COX-2 inhibition blunted the increase in iPGE_2_ in HK-2 cells under hypoxia (Fig. 3c). Interestingly, hypoxia also determined transient up-regulation of mPGES-1 protein expression (Fig. 3a, inset), but did not affect mPGES-1 mRNA expression (Fig. 3b, inset). These results indicated that increased expression of COX-2 and mPGES-1 is responsible for hypoxia-induced increase in iPGE_2_.

To further examine the contribution of transcriptional mechanisms to the increase in COX-2 protein expression under hypoxia, expression of COX-2 was assessed in HK-2 cells, which were pre-incubated with the transcription inhibitor actinomycin D before being subjected to hypoxia for 5 h. As shown in Fig. 3d, hypoxia-induced increase in COX-2 protein expression was prevented by actinomycin D. Furthermore, hypoxia also determined an increase in the activity of a COX-2 promoter construct previously transfected in HK-2 cells (Fig. 3d right). Taken together, the results shown in Fig. 3d suggest that transcriptional mechanisms contribute to hypoxia-induced increase in COX-2 protein expression. Nevertheless, there was a discrepancy between the timing of the activation of the COX-2 promoter and the timing of maximal COX-2 mRNA expression: while the transient increase in COX-2 mRNA expression was maximal after 2 h (Fig. 3b), the COX-2 promoter was activated after 3 h (Fig. 3d), which probably reflects the contribution of post-transcriptional mechanisms to the increase in COX-2 expression^42^. Accordingly, we speculate that COX-2 mRNA is stabilized under hypoxia, which would result in increased levels of COX-2 mRNA expression without any contribution (at that moment) of increased activity of the COX-2 promoter. However, it is also possible that a time lag between activation of the promoter and accumulation of measurable substrate may also contribute discrepancy between the timing of the activation of the COX-2 promoter and the timing of maximal COX-2 mRNA expression.

HIF-1 is a heterodimeric transcription factor composed of the oxygen-dependent α-subunit and the constitutively expressed β-subunit. Under hypoxia HIF-1α accumulates and enters the nucleus, where it generates the transcription factor HIF-1 after dimerizing with HIF-1β. HIF-1 then promotes the expression of its target genes by binding to hypoxia-responsive elements (HREs) present in their regulatory region and subsequently orchestrates cellular adaptive responses to combat hypoxia^43^. Given that hypoxia may activate COX-2 expression in a HIF-1-dependent manner through a functional HRE present in the COX-2 promoter sequence^12^, we examined the role of HIF-1α in hypoxia-induced increase in COX-2 expression. We also looked at the activity of a COX-2 promoter construct transfected in HK-2 cells. As shown in Fig. 3e, pre-incubation with the HIF-1α inhibitor YC-1, which blunted hypoxia-induced increase in HIF-1α expression and activity of a HRE reporter construct, resulted in prevention of the increase in both expression of COX-2 and activity of the COX-2 promoter construct in HK-2 subjected to hypoxia. In summary, the results are shown in Fig. 3a–e support the notion that HIF-1α-dependent transcriptional up-regulation of COX-2 is responsible for hypoxia-induced increase in iPGE_2_.

Although the consequences of the activation of HIF-1α on hypoxia-induced apoptosis are controversial (probably because they may be context-dependent^37^), we analyzed (in a preliminary manner) the role of HIF-1α in our experimental system. Since we have previously found that intervention in the COX-2/iPGE_2_/EP receptor pathway inhibits the increase in HIF-1α expression triggered by hypoxia^18,36^ we asked whether inhibition of HIF-1α results in prevention of hypoxia-induced HK-2 cell apoptosis. As shown in Fig. 3f, pre-incubation with YC-1, a HIF-1α inhibitor^44^, actually increased hypoxia-induced apoptosis. Therefore, it is unlikely that HIF-1α plays a role in the mechanism through which intervention in the COX-2/iPGE_2_/EP receptor pathway prevents the apoptosis of HK-2 cells under hypoxia.

## Discussion

Tissue hypoxia is thought to be critically relevant in the pathophysiology of both chronic kidney disease and acute kidney injury, being PTC the most susceptible portion of renal tubules against hypoxia. Here we examined the role of PGE_2_ in the damage inflicted by hypoxia to human PTC and found that increased production of iPGE_2_, originated from HIF-1α-dependent up-regulation of COX-2 gene expression and increased expression of mPGES-1, contributes to hypoxia-induced apoptotic cell death in HK-2 cells. Given that tubular hypoxia is a relevant factor for both acute and chronic kidney disease^1,2^, these results point out to prostaglandin transporter PGT as a new therapeutic target in renal diseases.

Hypoxia not only increases iPGE_2_ but also its release to the extracellular medium^18^ so that secreted (extracellular) PGE_2_ might also play a role in hypoxia-induced apoptosis (for instance, triggering apoptotic cascades through the canonical activation of EP receptors located at the cell membrane). Two previous studies have shown that cell death under hypoxia was also due to COX-2-dependent production of PGE_2_^13,45^. On the other hand, in certain cell types (fibroblasts, microglia, neurons, and acute lymphoblastic leukemia cell lines), PGE_2_-induced apoptosis is mediated by PGE_2_-dependent activation of EP-receptors^46–48^. We have found here that hypoxia-induced HK-2 cell apoptosis was prevented by antagonism of EP receptors as well (Fig. 2a, upper panel, right). But because EP receptors have been classically described as plasma membrane receptors^19^—so that they are inaccessible for iPGE2- we speculate that the EP receptors that mediate the apoptotic effect of iPGE_2_ in hypoxic HK-2 cells are a subset located intracellularly (i.e. iEP receptors).

In contrast to the role of iPGE2 in hypoxia-induced apoptosis in HK-2 cells, we found that only extracellular PGE_2_ mediates hypoxia/reoxygenation-induced HK-2 cell death because it was not prevented by inhibitors of PGT (Fig. 2f). Although the pathological mechanisms of cellular injury after hypoxia/reoxygenation are not completely understood, it is accepted that cell death largely occurs through the production of excessive reactive oxygen species (ROS). This is particularly true for HK-2 cells^49,50^. However, although hypoxia may also increase the production of ROS, to the best of our knowledge there is no evidence that ROS play a relevant in hypoxia-induced HK-2 cells death. This suggests that the lethal effects of hypoxia and hypoxia/reoxygenation in HK-2 cells are mediated by different mechanisms, which might explain why BG and BS are protective against hypoxia but not against hypoxia/reoxygenation. Consequently, inhibition of PGT might provide therapeutic benefits in PTC against hypoxia-induced cell injury but not against the deleterious effect of hypoxia/reoxygenation.

HIF-1α-dependent up-regulation of COX-2 was a critical event for hypoxia-induced HK-2 cell apoptosis (Fig. 3e). However, our observation that HIF inhibitor YC-1 enhanced apoptotic cell death (Fig. 3f) is puzzling, although previous reports have already shown similar results^51,52^ One possible explanation is a hypothetical scenario in which HIF-1α not only mediates the pro-apoptotic up-regulation of COX-2 but also the up-regulation of protective molecules. Indeed, HIF-1-regulated expression of protective molecules against hypoxia such as long non-coding RNA DARS-AS1^5^ and microRNA-210^53^ has been specifically found in HK-2 cells. However, although these pieces of evidence support our hypothetical scenario, specific experiments should be performed to confirm it.

Investigation of the response of renal cells to hypoxia may improve our understanding of renal pathology. We have studied, although in a preliminary manner, the role of an increase in the pro-apoptotic protein Bax to anti-apoptotic protein Bcl-2 expression ratio in hypoxia-induced apoptosis in HK-2 cells. Our results indicated that inhibition of PGT did not result in changes compatible with an anti-apoptotic shift in the balance between the expression of Bcl-2 and Bax (Fig. 2e), which does not support a role of these proteins in iPGE_2_-dependent HK-2 cell death under hypoxia. To the best of our knowledge there are only two studies on the implication of the Bcl-2/Bax axis in hypoxia-induced apoptosis in PTC and both of them involved anoxic^54^ or nearly anoxic O_2_ concentrations^53^. When PTC were exposed to 0.2% O_2_, Bax expression remained unchanged and apoptosis was linked to decreased Bcl-2 expression^6^. However, expression of Bcl-2 was unaffected by 1% O_2_ despite the presence of apoptosis^6^, which is coincident with our results. Therefore, other mechanisms that do not necessarily involve quantitative changes in Bcl-2 or Bax expression should be considered: for instance, induction of apoptosis in cultured glioma cells by iPGE_2_ only requires its physical association with Bax, which triggers the translocation of Bax to mitochondria^20,22^. Thus, the mechanism through which iPGE_2_ contributes to HK-2 cell apoptosis deserves further studies.

Our group has found that a COX-2-dependent increase in iPGE_2_ mediates apoptotic death in HK-2 cells exposed to cisplatin^7^, apoptotic bodies^55^ and hypoxia (our current results). Because PGE_2_ is rapidly released to the outside the cell after being synthesized^20^, its inward transport by PGT plays a critical role in iPGE_2_-induced apoptosis in HK-2 cells. Therefore, whenever that PTC damage is mediated by iPGE_2_, treatment with inhibitors of PGT might be a useful therapeutic approach with potential applications such as prevention of cisplatin-induced PTC damage, inhibition of the propagation of tubular injury through apoptotic bodies or limitation of the deleterious effect of hypoxia on PTC.

## Supplementary Information

Supplementary Information.

## Data Availability

The data sets used and/or analyzed during the current study are available from the corresponding author on reasonable request.
