# Supplementary material for: Intracellular prostaglandin E2 contributes to hypoxia-induced proximal tubular cell death
Source: Sci Rep. 2021 Mar 29;11:7047. doi: 10.1038/s41598-021-86219-w (PMC8007803; doi:10.1038/s41598-021-86219-w)

INTRACELLULAR PROSTAGLANDIN E2 CONTRIBUTES TO HYPOXIA-INDUCED PROXIMAL TUBULAR CELL DEATH.

Short title: INTRACELLULAR PGE$_2$ IN HYPOXIC CELL DEATH

Coral García-Pastor*, Selma Benito-Martínez*, Ricardo J. Bosch*, Ana B. Fernández-Martínez**[1], Francisco J. Lucio-Cazaña*[1]

*Departamento de Biología de Sistemas, Universidad de Alcalá, Alcalá de Henares, Madrid

** Departamento de Biología, Universidad Autónoma de Madrid, Madrid

[1] These authors contributed equally to this work

Address correspondence to:

Ana Belén Fernández-Martínez, Ph. D.

Departamento de Biología

Universidad Autónoma de Madrid

28049 Madrid

Phone: +34 91 497 8142

e-mail: anab.fernandez@uam.es

Supplementary Information File

1. Treatment with BG does not modify cell shape: phalloidin (green)/DAPI (blue) staining

2. a) Full length blot corresponding to the inset in Fig 2 a, lower panel, left: PGT and β-actin; b) Full length blot corresponding to the inset in Fig. 2 b: 15-PGDH and β -actin; c) Full length blots corresponding to Fig 2 e: Bcl-2, Bax and β-actin; d) Kinetics of cell death (trypan blue dye exclusion test) during reoxygenation after 24 h under hypoxia (1% O_2) and absence of effect of PGT inhibitor bromocresol green (BG). Cells were pre-incubated for 1 h with 50 μM BG inhibitors of the pathway. Each bar represents the mean ±SD of 3 different experiments *$P < 0.01$ vs control.*

3. a) Full length blot corresponding to Fig. 3 a (COX-2 and β-actin) and its inset (mPGES-1 and β-actin); b and c) Full length blots corresponding to Fig. 3 d and e: COX-2 and β-actin; d) Full length blot corresponding to the inset in Fig. 3 f: HIF-1α and β-actin

Figure suplementary 1

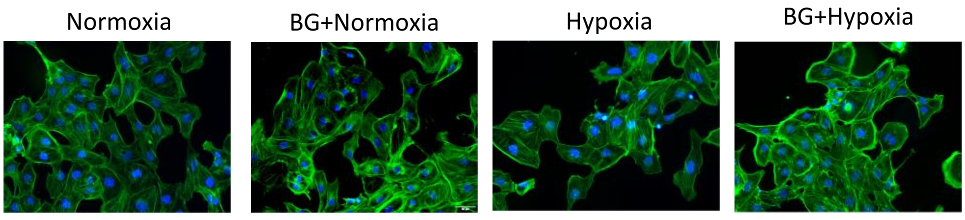

Figure suplementary 2

a)

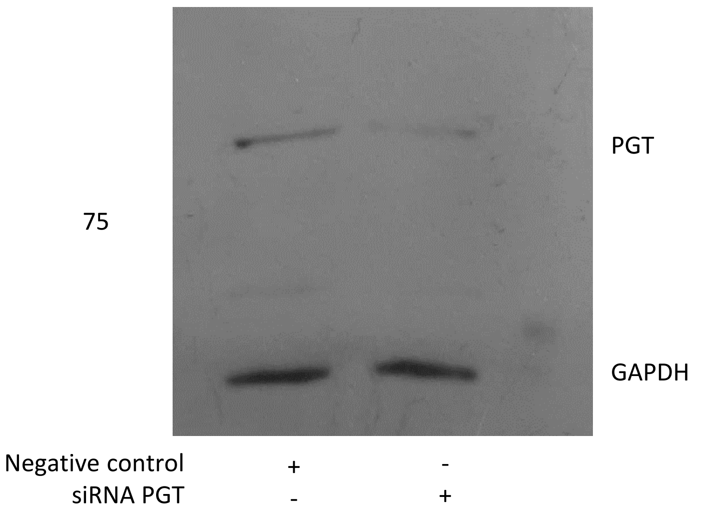

b)

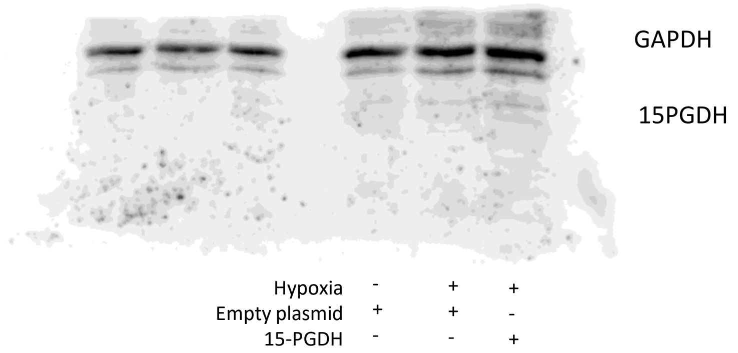

c)

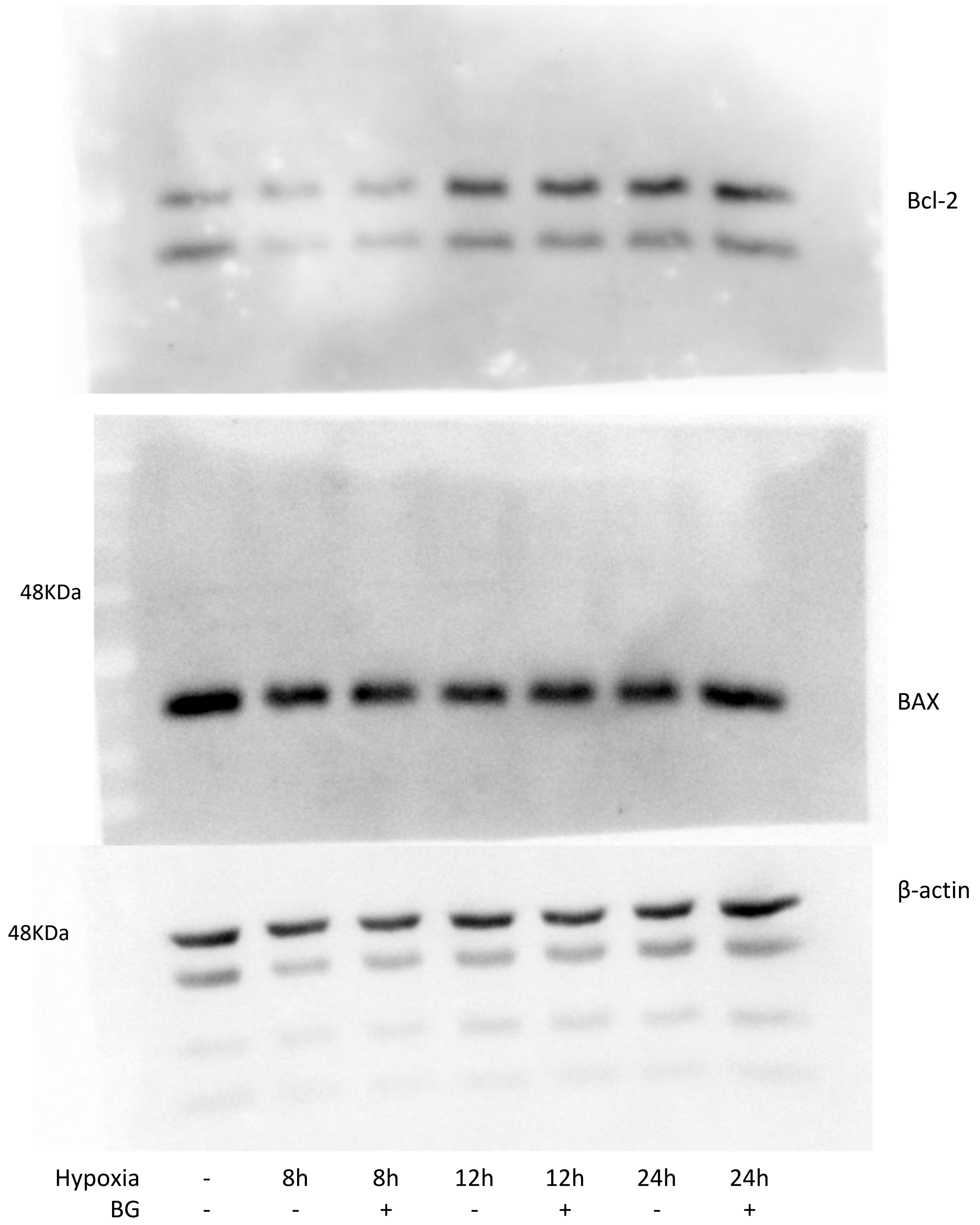

d)

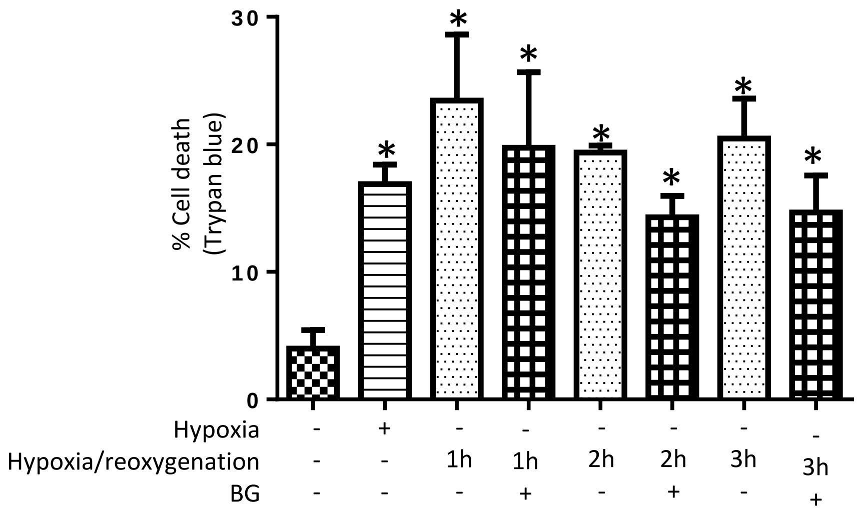

Figure suplementary 3

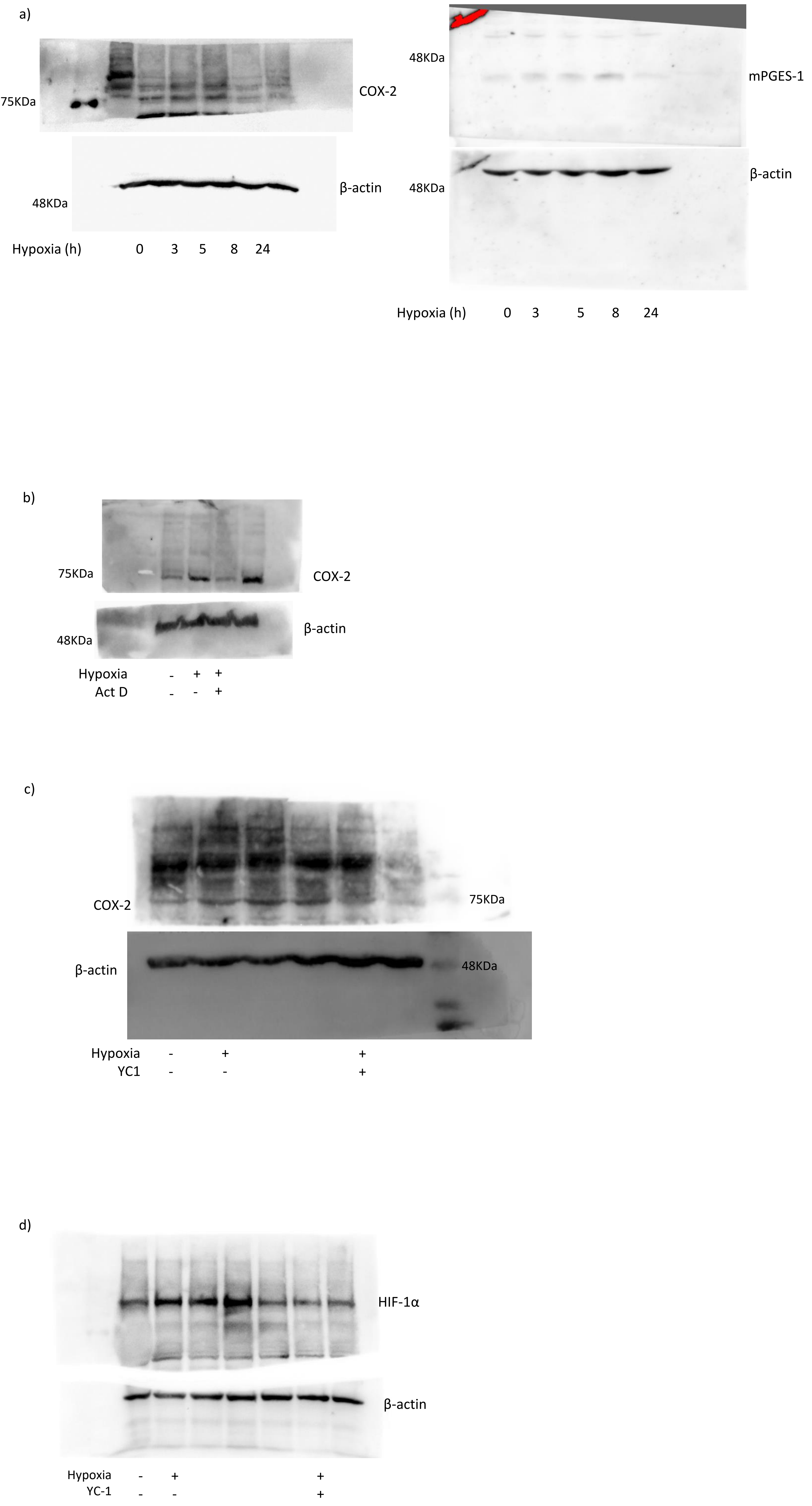

Supplement: Supplementary file 1 — Supplementary Information. [file 41598_2021_86219_MOESM1_ESM.pdf]
